# Georgia State Commission on Tuberculosis

**Published:** 1904-12

**Authors:** Chas. Hicks, Bernard Wolff

**Affiliations:** Dublin, Chairman; Permanent Secretary, Atlanta


### GEORGIA STATE COMMISSION ON TUBERCULOSIS.

DR. CHAS. HICKS, DUBLIN, Chairman.
DR. BERNARD WOLFF, PERMANENT SECRETARY, Atlanta.

| MEMBERS FROM STATE AT LARGE. | MEMBERS FROM CONGRESSIONAL DIST. |
|---|---|
| Dr. Chas. Hicks, Dublin, | 1st. Dr. E. R. Corson, Savannah, |
| Dr. Louis H. Jones, Atlanta, | 2d. Dr. O. B. Bush Camilla, |
| Dr. Earnest P. Ham, Gainesville, | 3d. Dr. Alex. Mack, Hawkinsville, |
| Dr. P. L. Hilsman, Albany, | 4th. Dr. H. R. Slack, LaGrange, |
| Dr. Jos. R. Burdett, Tennille, | 5th. Dr. Bernard Wolff, Atlanta, |
| Dr. Pryor W. Fitts, Greenville, | 6th. Dr. George F. Alexander, Forsyth, |
| Dr. L. G. Hardman, Commerce, | 7th. Dr. Thomas R. Garlington, Rome, |
| Dr. L. J. Belt, Millen, | 8th. Dr. Henry E. Thornton, Hartwell, |
| Dr. J. L. Walker, Waycross, | 9th. Dr. Jefferson Davis, Toccoa, |
| Dr. S. A. Brown, Dalton. | 10th. Dr. Thomas Coleman, Augusta, |
| | 11th. Dr. S. E. Sanchez, Barwick. |

DEAR DOCTOR: —

Please fill out this report to the best of your knowledge and return it to me at your earliest convenience. The report is confidential and intended solely for statistical purposes.

Yours fraternally,

...................., M. D., ................, Ga.

Member Georgia State Commission on Tuberculosis for the ....................Congressional District.

REPORT ON CASES OF TUBERCULOSIS.

From.............................., Address. ..................................

| No. of Cases. | Age. | | | | | | | Sex. | No. of Whites. | Civil Condition. | Occupation. |
|---|---|---|---|---|---|---|---|---|---|---|---|
| | Under 10 Years. | From 10 to 20. | 20 to 30. | 30 to 40. | 40 to 50. | 50 to 60. | Over 60. | | | | |
| 1. How many cases of Consumption have occurred in your practice during the last twelve months............. | | | | | | | | Males. | | Married.<br>Single. | |
| 2. How many have died ?............... | | | | | | | | Females. | No. of Colored | Widow'd | |

3. How many cases have been contracted in your locality and how many imported from elsewhere ..........................................................

[ (a) Local ......................... (b) Imported.................]

4. Have any measures been taken in your locality to prevent the spread of the disease ?.............................................

5. Do you wish the State to take measures for the prevention of the disease ?.................................

The above cases have come under my observation during the year ending.................................. 190—.

................................................ M. D.

Address.................................. Date................................

The foregoing is the form of blank adopted by the Georgia State Commission on Tuberculosis for collecting statistics. The Commission wishes to make its final report to the next General Assembly to be as full and accurate as possible and to this end it is desired that every medical man in Georgia shall receive one of these blanks and his individual report obtained. If any one has been overlooked in the distribution of these blanks by his District Commission he is earnestly requested to fill out the above form, cut it out and return it to the secretary.

